# Neuroprotective Effects of Diets Containing Olive Oil and DHA/EPA in a Mouse Model of Cerebral Ischemia

**DOI:** 10.3390/nu11051109

**Published:** 2019-05-18

**Authors:** Rafael Gonzalo-Gobernado, María Irene Ayuso, Loredana Sansone, Juan José Bernal-Jiménez, Víctor Darío Ramos-Herrero, Enrique Sánchez-García, Teresa L. Ramos, Rocío Abia, Francisco J. G. Muriana, Beatriz Bermúdez, Joan Montaner

**Affiliations:** 1 Neurovascular Research Group, Institute of Biomedicine of Seville, IBiS, Hospital Universitario Virgen del Rocío, Av. Manuel Siurot s/n, 41013 Seville; Spain; rgonzalo-ibis@us.es (R.G.-G.); loredana.sansone@hotmail.it (L.S.); jbernalto@gmail.com (J.J.B.-J.); vramos-ibis@us.es (V.D.R.-H.); kekoshan@hotmail.com (E.S.-G.); jmontaner-ibis@us.es (J.M.); 2 Laboratory of Cell Therapy and New Therapeutic Targets in Onco-Hematology, Institute of Biomedicine of Seville, IBiS, Hospital Universitario Virgen del Rocío, Av. Manuel Siurot s/n, 41013 Seville, Spain; teresaclramos@gmail.com; 3 Laboratory of Cellular and Molecular Nutrition, Instituto de la Grasa, CSIC, Ctra. de Utrera Km. 1, 41013 Seville, Spain; abia@ig.csic.es (R.A.); muriana@ig.csic.es (F.J.G.M.); 4 Department of Cellular Biology, School of Biology, University of Seville, Av. de la Reina Mercedes 6, 41012 Seville, Spain; bbermudez@us.es; 5 Department of Neurology, Hospital Universitario Virgen Macarena, Av. Doctor Fedriani 3, 41007 Seville, Spain

**Keywords:** cerebral ischemia, stroke, middle cerebral artery occlusion (MCAo), omega-3 fatty acids, DHA, EPA, olive oil, MUFA, PUFA, SFA, neuroprotection, neurological function, behaviour

## Abstract

Stroke is one of the leading causes of death worldwide and while there is increasing evidence that a Mediterranean diet might decrease the risk of a stroke, the effects of dietary fat composition on stroke outcomes have not been fully explored. We hypothesize that the brain damage provoked by a stroke would be different depending on the source of dietary fat. To test this, male C57BL/6J mice were fed for 4 weeks with a standard low-fat diet (LFD), a high-fat diet (HFD) rich in saturated fatty acids (HFD-SFA), an HFD containing monounsaturated fatty acids (MUFAs) from olive oil (HFD-OO), or an HFD containing MUFAs from olive oil plus polyunsaturated fatty acids (PUFAs) docosahexaenoic acid/eicosapentaenoic acid (DHA/EPA) (HFD-OO-ω3). These mice were then subjected to transient middle cerebral artery occlusion (tMCAo). Behavioural tests and histological analyses were performed 24 and/or 48 h after tMCAo in order to elucidate the impact of these diets with different fatty acid profiles on the ischemic lesion and on neurological functions. Mice fed with HFD-OO-ω3 displayed better histological outcomes after cerebral ischemia than mice that received an HFD-SFA or LFD. Furthermore, PUFA- and MUFA-enriched diets improved the motor function and neurological performance of ischemic mice relative to those fed with an LFD or HFD-SFA. These findings support the use of DHA/EPA-omega-3-fatty acid supplementation and olive oil as dietary source of MUFAs in order to reduce the damage and protect the brain when a stroke occurs.

## 1. Introduction

Stroke is one of the leading causes of death worldwide [1] and is a major cause of disability in adults [2]. A stroke occurs when a cerebral blood vessel is disrupted and as a result, the blood supply to a region of the brain is cut-off [3]. The sequence of events responding to ischemia is known as the ischemic cascade, which includes glutamate release, calcium influx, oxidative stress, inflammation, and ultimately, apoptosis, the irreversible loss of cell viability and neuronal death [4,5]. In addition, comorbid conditions, the accumulation of risk factors for stroke, and a western diet, can all exacerbate the subsequent ischemic damage [6,7].

High-fat diets (HFDs) are known to induce metabolic changes and obesity, and can promote chronic inflammation [8]. Diet-induced obesity and the associated metabolic disturbances (e.g., hyperlipidemia) have also been proposed as risk factors for cardiovascular diseases and stroke [9]. HFDs enhance inflammatory responses, induce neuroinflammation, and are associated with worse outcomes in experimental studies [7,10,11]. Consequently, approaches that aim to disrupt the ischemic cascade at early stages, thereby minimizing the associated oxidative stress and inflammation, might help to prevent the progression of damage and improve functional outcomes after the insult. Diet is a key factor in vascular diseases and clinical studies suggest that type of diet contributes to neurovascular disease in our elders, with a healthy diet lowering cardiovascular and stroke risk (Prevención con Dieta Mediterránea, PREDIMED study, [12]). Moreover, most stroke patients appear to have an unhealthy diet [13], which, independently of pre-existing co-morbid conditions, is an important factor influencing the outcome of ischemic stroke [7,14].

The nutritional qualities of each individual diet are determined by their specific components. The beneficial effects of adhering to the Mediterranean diet are widely known, a diet characterized by the presence of beneficial bioactive compounds, such as monounsaturated and polyunsaturated fatty acids (MUFAs and PUFAs) or polyphenols [15]. Indeed, elements of this diet modify the responses of different targets to inflammatory or oxidative mediators [16]. The Mediterranean diet is also characterized by a regular intake of fish [17]. Interestingly, it is well-known that moderate fish and seafood consumption influences cerebrovascular mortality [18], and it is associated with a lower incidence of subclinical brain infarcts [19]. Fish oil is a significant source of omega-3 fatty acids, mainly eicosapentaenoic acid (EPA) and docosahexaenoic acid (DHA), the consumption of which is thought to have cardiovascular benefits and is associated with a lower risk of death from cardiovascular disease [20]. Olive oil is rich in oleic acid, a non-essential monounsaturated fatty acid found in high amounts in vegetable oils. Olive oil consumption is associated with a lower risk of ischemic stroke, which suggests it also has neuroprotective effects [12]. In fact, olive oil protects against neuronal cell death in rodent cerebral ischemia models [21], ameliorating brain injury [22,23].

Diet not only serves as a vascular risk factor, but we propose that if ischemic injury occurs, the evolution and the extent of damage would differ depending on the diet of the individual. However, the effects of dietary fat composition on stroke damage have yet to be explored in depth. We hypothesized that the damage to the brain would be different depending on the source of dietary fat. Consumption of an HFD enriched in omega-3 fatty acids and MUFAs prior to the induction of cerebral ischemia may have beneficial effects, reducing ischemic injury and neurological deficits. Thus, the main goal of this study was to determine the neuroprotective effect of pre-treatment with an HFD enriched in DHA/EPA and olive oil in a mouse model of transient focal ischemia produced by distal occlusion of the middle cerebral artery (MCA).

## 2. Materials and Methods

### 2.1. Animals

Male C57BL/6J mice were purchased from Charles River and then housed in a 12 h light/dark cycle, in a humidity and temperature (22 ± 2 °C)-controlled environment with ad libitum access to food and water. The study was carried out in accordance with the Declaration of Helsinki, and the protocol was approved by the Ethics Committee of Hospitales Universitarios Virgen Macarena y Virgen del Rocío (Project identification code: 11-09-15-323). All procedures were performed in compliance with the ARRIVE guidelines and Spanish legislation, and in accordance with EU Directives.

### 2.2. Diets

All of the diets studied here were kindly designed by Dr Francisco J. G. Muriana from the Laboratory of Cellular and Molecular Nutrition, Instituto de la Grasa, CSIC, Seville, Spain. The diets were prepared at Panlab Laboratories (SAFE, Augy, France) and provided to the mice as pellets. All of the diets were based on the standard rodent A04-10 diet, containing 0.01% cholesterol, 20 mg per kg BHT, and 3% binder. The diets used in this study were: a standard normal-fat A04-10 diet (low-fat, low-cholesterol diet (LFD)) containing 3% of energy as fat, or three different high-fat, low-cholesterol custom diets (HFDs), in which 24% of the energy was supplied through fat. The three different HFDs were prepared by replacing the fat source from the A04-10 diet with: cow’s milk cream rich in saturated fatty acids (SFAs, 24% energy, high-fat diet rich in saturated fatty acids (HFD-SFA)), refined olive oil rich in MUFAs (24% energy, high-fat diet rich in olive oil (HFD-OO)), or refined olive oil rich in MUFAs (23% energy) plus the omega-3 PUFAs, DHA and EPA, in the form of ethyl esters (1% energy, HFD-OO-ω3). The cow’s milk cream provided an additional 0.006% cholesterol by weight, while all of the diets contained equal proportions of protein (19.5% energy) and carbohydrates were used to adjust the total energy content (Table 1).

### 2.3. Fatty Acid Composition of Dietary Fats

The fatty acid composition of dietary fats (cow’s milk cream rich in SFAs, refined olive oil rich in MUFAs, and refined olive oil plus eicosapentaenoic acid (EPA) and docosahexaenoic acid (DHA) rich in MUFAs and omega-3 long-chain PUFAs) was determined by the method described in EEC/796/2002 [24]. This involved using a gas chromatography system (HP-5890, Hewlett-Packard, Palo-Alto, CA, USA) equipped with a flame ionization detector and a SP-2380 capillary column (Supelco, Bellefonte, PA, USA, 30 m × 0.32 mm) packed with cyanopropyl siloxane (0.25 μm). The initial column temperature was 165 °C, which was held for 10 min and then programmed to rise from 165 °C to 200 °C, at 1.5 °C min^−1^. The injector and detector temperatures were 250 °C, with H_2_ as the carrier gas. The fatty acid composition of the dietary fats in each customized diet (HFD-SFA, HFD-OO and HFD-OO-ω3) is shown in Table 2 and was similar to that described in [25].

### 2.4. Experimental Design

**Figure 1:**
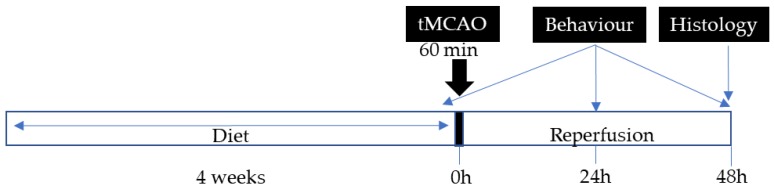
Representative scheme showing the procedure followed to study the effect of HFDs with distinct fatty acid compositions on mice subjected to transient middle cerebral artery occlusion (tMCAo).

The experimental protocol is shown in Figure 1. Six-week-old C57BL/6J mice were randomly allocated to the four experimental groups and fed for 4 weeks with LFD, HFD-SFA, HFD-OO, or HFD-OO-ω3 diets before experimental cerebral ischemia was induced. The food pellets were replaced twice a week in order to prevent lipid peroxidation and body weight was monitored weekly throughout the experiment. Food intake was monitored for the first 10 days of the experiment. All of the researchers remained blind to the experimental treatments throughout the experiment, as well as the subsequent procedures and analyses.

### 2.5. Middle Cerebral Artery Occlusion

Transient occlusion of a distal branch of the middle cerebral artery (tMCAo) was induced in male C57BL/6J mice (10-week-old), as described previously [26,27]. Briefly, mice were anesthetized with 4% isoflurane for induction and 1.5–2% isoflurane for maintenance (in 79% N_2_/21% O_2_), and after drilling a small hole in the temporal bone, the middle cerebral artery (MCA) was compressed for 60 min with a 30-G needle using a micromanipulator. During surgery, body temperature was maintained at 37.0 ± 0.5 °C using a homoeothermic blanket, and cerebral blood flow (CBF) was monitored using laser-Doppler flowmetry to confirm MCA occlusion. Buprenorphine (0.05–0.1 mg/kg) was administered subcutaneously immediately before the procedure. A total of 42 mice were subjected to tMCAo according to the study design, having performed a power analysis to determine the sample size (significance level set at 0.05, the power set at 0.8 (80%)) that identified the need for 8 mice to be included in each experimental group. Additional mice were allocated taking into account mortality and technical issues that arise during the experiments based on experience. Surgical inclusion criteria were: a reduction in blood flow to <25% of baseline value during the ischemia period, and a recovery of 75% of the baseline value in the reperfusion period. Mice that met the following criteria were excluded: (i) mice that failed to meet the inclusion criteria explained above (3 mice); and (ii) mice that died during the induction of middle cerebral artery occlusion (MCAo) (3 mice).

### 2.6. Infarct Volume, Infarct Severity, and the Evaluation of Haemorrhagic Transformation

The size of infarction was evaluated 48 h after tMCAo using 2,3,5-tetrazolium chloride (TTC) staining [28]. The mice were sacrificed by transcardial perfusion with ice-cold saline under deep anaesthesia. Brains were removed and cut into 1-mm-thick coronal sections and stained with 2.5% of TTC in saline for 20 min at room temperature. The infarct areas and differences in grayscale were measured using the Image-J software (National Institutes of Health, Bethesda, MD, USA), blind to the experimental procedures. The infarct volume was determined by linear integration of the lesion areas measured and the distances across the sections. In order to avoid any effects of brain edema, the infarct area was corrected by the ratio of the area of the ipsilateral and the contralateral hemisphere. Edema was calculated using the following formula: Edema (%) = ((Ipsilateral Hemispheric Volume − Contralateral Hemispheric Volume) × 100)/Contralateral Hemispheric Volume.

The infarct severity was evaluated by performing a grayscale analysis of the TTC-stained brain sections, as described in [29,30], with slight modifications. The mean grayscale of the lesioned area of the hemisphere and the contralateral area were analysed in each mouse using the Image-J software, and the difference of both values was determined and expressed as an absolute value. Higher values were associated with tissue damage, and values close to zero indicate that there is no difference between the areas of the ischemic hemisphere analysed and their respective areas in the contralateral hemisphere. Haemorrhagic transformation (HT) was assessed macroscopically on digital images from coronal slices and classified as described previously [31,32]. Macroscopic haemorrhages were classified into five groups: (0) no haemorrhages, (1) haemorrhagic infarction type 1 (HI-1), (2) haemorrhagic infarction type 2 (HI-2), (3) parenchymal haemorrhage type 1 (PH-1), (4) parenchymal haemorrhage type 2 (PH-2).

### 2.7. Neurobehavioural Tests

Neurobehavioural tests to evaluate motor function and neurological outcomes were performed before surgery and/or 24 or 48 h after tMCAo. The tests were carried out by a researcher blind to the experimental conditions.

#### 2.7.1. Grip Strength Test

The grip strength test is designed to assess the maximum force in the mouse’s forelimbs using a metal grid connected to a force sensor (Bioseb, Vitrolles, France). The test was performed as described previously [26,33,34] and a total of 6 trials were carried out in each test session, calculating the strength (in grams) as the mean of these trials.

#### 2.7.2. Neurological Scale

Neurological scales are used to evaluate motor, sensory, reflex, and balance alterations induced by cerebral ischemia. The neurological scale used in this work was that adapted from Bederson´s scale [35] and the modified Neurological Severity Score (mNSS) [36] with several modifications, whereby the higher the score, the more severe the injury. The items evaluated were scored as follows: (1) dorsal kyphosis 1 point (p), (2) static torsion (head and body asymmetry when the animal is raised by its tail) 1p, (3) dynamic torsion (head and body asymmetry after gently shaking when the animal is raised by its tail) 1p, (4) forelimb flexion 2p, (5) absence of a forelimb grasping reflex 1p, (6) absence of a vibrissae reflex 2p, (7) absence of the righting reflex 3p, (8) inability to walk straight 1p, (9) circling 2p, (10) 5–10% decrease in forelimb strength 1p, 11–20% 2p, and a 21% or higher decrease 3p. The maximum score in this scale is 17 points.

#### 2.7.3. Footprint Test

Footprint analysis is a simple method used to measure gait impairment in mice. The test was performed 48 h after tMCAo as described in [37,38], with slight modifications. Briefly, the mouse’s fore and hind paws were painted with non-toxic blue or red water-based paint, respectively. The mice were allowed to walk freely along a corridor (60 × 10 × 7.5 cm) towards a dark box at the end of the corridor, after lining the corridor with strips of white paper. All mice were habituated to the corridor over three training runs prior to testing and six different aspects of gait were then measured: fore and hind limb stride length, front and base width, overlap, stance, steps/m, and stride coordination (to determine the coordination between strides, the length of the shortest stride was subtracted from the length of the longest stride, as described in [39]). For each parameter, three values were obtained from each test session, excluding footprints made at the beginning and the end of the run, and footprints made by mice that walked along the corridor at an irregular speed. The footprints were scanned, and the distances were measured using Image Pro Plus software. Each parameter was calculated as the mean of each set of three values.

### 2.8. Determination of Antioxidant Enzyme Activity

Blood was collected from the right ventricle of the heart using 21-G needles and EDTA tubes when the mice were sacrificed 48 h after tMCAo. Plasma samples were obtained by centrifugation at 1300 g for 10 min at 4 °C and they were stored at −80 °C until use.

Catalase (CAT), glutathione reductase (GR) and glutathione peroxidase (GPx) activity was determined to assess the oxidative stress in plasma samples. CAT (U/mg protein) was determined following the method described by Cohen and Somerson [40] and by using a microplate multilabel reader (Victor 3, PerkinElmer, MA, USA). GR and GPx (mU/mg protein) were measured using a kit purchased from Randox Laboratories Ltd. (Crumlin, UK) (Cat. No. GR2368 and RS505, respectively) and a computer process-controlled multichannel auto-analyser (Cobas Integra 400, Roche Diagnostic, (Barcelona, Spain). These analyses were performed at the Laboratory of Ecophysiology of the Estación Biológica de Doñana, Spain (EBD-CSIC).

### 2.9. Statistical Analysis

All statistical analyses were performed by researchers who were blind to the experimental treatments, using GraphPad Prism software (San Diego, CA, USA). The results are expressed as the mean ± standard error of the mean (SEM) (quantitative data) or median ± interquartile range (categorical data) of (*n*) mice. Statistical analyses of the histopathological studies or footprint test, and grip strength test were performed using one-way or two-way ANOVA respectively, followed by a Bonferroni’s post-hoc test when the analysis of variance was significant. A Kruskal–Wallis test, followed by Dunn’s multiple comparisons test when Kruskal–Wallis test was significant, was used to analyse the categorical data from the neurological severity score and the haemorrhagic transformation score. Differences were considered significant when *p* ≤ 0.05.

## 3. Results

### 3.1. Animal Growth and Food Intake

**Figure 2:**
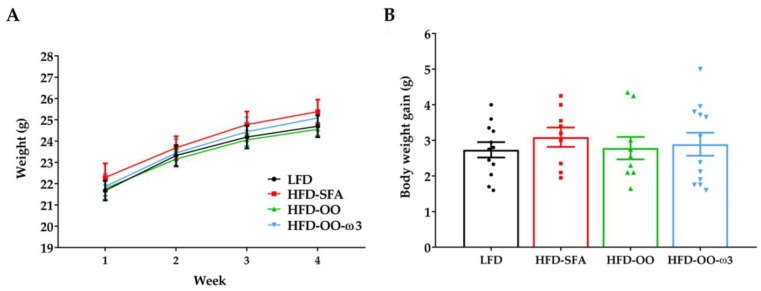
(**A**) Body weight monitoring and (**B**) Body weight gain. Experimental diets: LFD (Black), HFD-SFA (Red), HFD-OO (Green), and HFD-OO-ω3 (Blue). No significant differences between the groups were found after ANOVA. The results represent the mean ± standard error of the mean (SEM) of 9 to 12 animals. Low-fat diet (**LFD**)**,** high-fat diet rich in saturated fatty acids (**HFD-SFA**)**,** high-fat diet rich in olive oil (**HFD-OO**) and high-fat diet rich in olive oil plus DHA and EPA (**HFD-OO-ω3**).

The growth of the mice was analysed while they received each diet by weighing them weekly. After four weeks on the experimental dietary regime, no differences in body weight (Figure 2A) or gains in body weight (Figure 2B) were found between the mice in the LFD, HFD-SFA, HFD-OO, or HFD-OO-ω3 groups.

To analyse the adherence to the different diets, food intake was evaluated over the first 10 days of the experiment. Animals in the HFD-SFA, HFD-OO, and HFD-OO-ω3 groups seemed to have a higher mean food intake those in the LFD group, yet no significant differences were observed between the four experimental diets studied (Table 3).

### 3.2. Cerebral Blood Flow (CBF)

**Figure 3:**
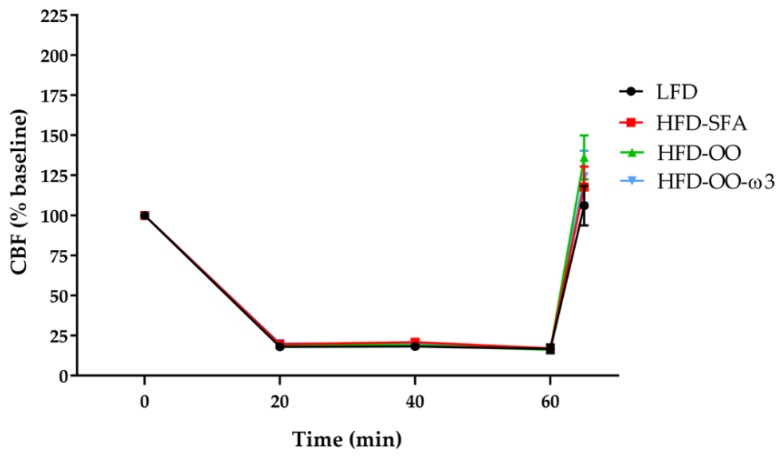
Representation of cerebral blood flow (CBF) traces recorded by laser Doppler flowmetry during the tMCAo procedure. CBF values were recorded continuously during the procedure, analysing the following time intervals: baseline (0 min), 0–20 min, 20–40 min, 40–60 min, and the onset of reperfusion at 60–65 min. Groups: LFD (Black), HFD-SFA (Red), HFD-OO (Green) and HFD-OO-ω3 (Blue). No significant differences between the groups were found (2-way ANOVA), the results represent the mean ± SEM of 8 to 10 animals. Low-fat diet (**LFD**), high-fat diet rich in saturated fatty acids (**HFD-SFA**), high-fat diet rich in olive oil (**HFD-OO**), and high-fat diet rich in olive oil plus DHA and EPA (**HFD-OO-ω3**).

CBF was registered by laser doppler flowmetry during tMCAo and no significant differences in CBF traces were found between ischemic mice fed with the LFD, HFD-SFA, HFD-OO, or HFD-OO-ω3 diets (Figure 3).

### 3.3. A High-Fat Diet Enriched in DHA/EPA and MUFAs from Olive Oil Reduces the Infarct Area in Mice Subjected to Transient Focal Cerebral Ischemia

**Figure 4:**
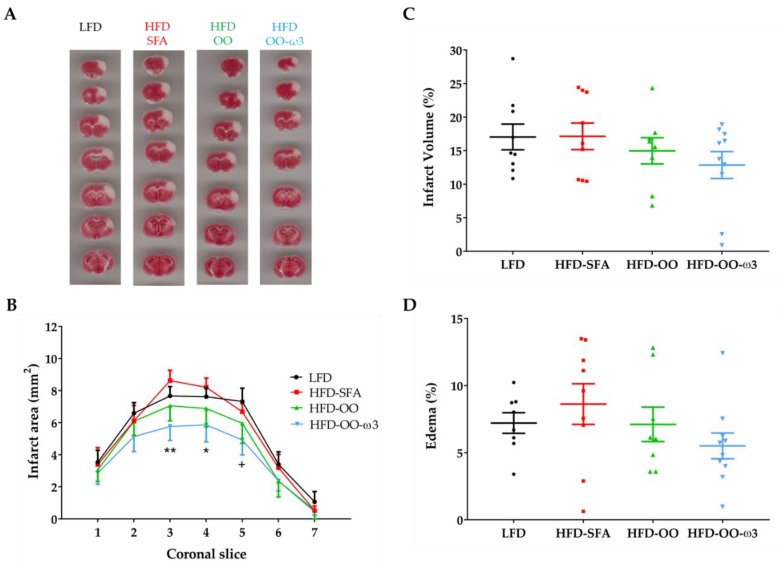
HFD-OO-ω3 diet reduces the infarct area in an experimental model of focal cerebral ischemia. Groups: LFD (Black), HFD-SFA (Red), HFD-OO (Green), and HFD-OO-ω3 (Blue). (**A**) Representative images of TTC staining. Seven coronal slices along the rostro-caudal axis are shown, from coordinates bregma AP: +2.80 to bregma AP: −2.46 (Franklin and Paxinos atlas [41]). (**B**) The HFD-OO-ω3 diet reduced the infarct area compared to HFD-SFA and LFD. (**C**,**D**) show the Infarct volume and edema analyses, respectively. These data reflect a slight decrease in both parameters, yet no significant differences were found. The results represent the mean ± SEM of 8 to 10 animals: * *p* ≤ 0.05, ** *p* ≤ 0.01 versus HFD-SFA and ^+^ *p* ≤ 0.05 versus LFD (ANOVA + Bonferroni post-hoc). Low-fat diet (**LFD**), high-fat diet rich in saturated fatty acids (**HFD-SFA**), high-fat diet rich in olive oil (**HFD-OO**), and high-fat diet rich in olive oil plus DHA and EPA (**HFD-OO-ω3**).

To study the effect of the diets on the consequences of tMCAo, the infarct area, infarct volume, and edema were evaluated by TTC staining and image analysis performed on coronal slices of ischemic brains 48 h after MCA occlusion (Figure 4). The mouse model of transient cerebral ischemia used in this study provokes a cortical lesion that is limited to the territory irrigated by the left MCA (Figure 4A). Significantly, the size of the ischemic area in mice fed with HFD-OO-ω3 was smaller than that in the HFD-SFA and LFD groups (Figure 4A,B), indicating that the diet enriched in DHA/EPA and MUFAs from olive oil exerted a neuroprotective effect. The analyses of the infarct volume (Figure 4C) and edema (Figure 4D) also appeared to reflect a slight decrease in both parameters in the HFD-OO-ω3-fed animals, although the changes observed were not statistically significant.

### 3.4. Beneficial Effects of Dietary Intervention with DHA+EPA and Olive Oil-Enriched Food on Brain Infarct Severity

**Figure 5:**
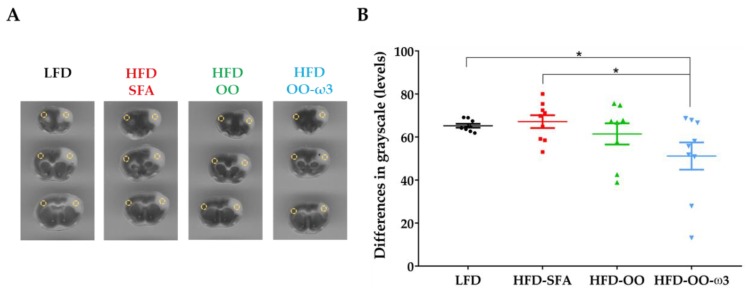
HFD-OO-ω3 diet improves infarct severity in mice subjected to transient focal cerebral ischemia. Groups: LFD (Black), HFD-SFA (Red), HFD-OO (Green), and HFD-OO-ω3 (Blue). (**A**) Representative grayscale images of TTC staining to determine infarct severity. Three coronal sections along the rostro-caudal axis are shown, as well as the ipsilateral and contralateral ROI probes used in the analysis (yellow circles). (**B**) HFD-OO-ω3 diet improved brain infarct severity compared to HFD-SFA and LFD. The results represent the mean ± SEM of 8 to 9 animals: * *p* ≤ 0.05 (ANOVA + Bonferroni post-hoc). Low-fat diet (**LFD**), high-fat diet rich in saturated fatty acids (**HFD-SFA**), high-fat diet rich in olive oil (**HFD-OO**), and high-fat diet rich in olive oil plus DHA and EPA (**HFD-OO-ω3**).

Infarct severity was evaluated by performing a grayscale analysis of the TTC-stained brain sections obtained 48 h after mice were subjected to tMCAo. Mice fed with HFD-OO-ω3 displayed a significant reduction in the grey scale intensity compared to animals fed with HFD-SFA and LFD (Figure 5A,B), revealing a neuroprotective effect of the diet enriched in DHA+EPA and olive oil.

### 3.5. Haemorrhagic Transformation

**Figure 6:**
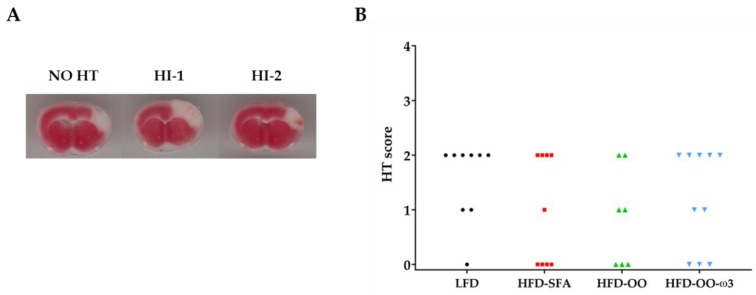
Haemorrhagic transformation analysis. Groups: LFD (Black), HFD-SFA (Red), HFD-OO (Green), and HFD-OO-ω3 (Blue). (**A**) Representative images of the classes of macroscopic haemorrhages found during the analysis: no haemorrhages (NO HT), haemorrhagic infarction type 1 (HI-1), haemorrhagic infarction type 2 (HI-2). (**B**) Haemorrhagic transformation score. No significant differences were found between the groups (Kruskal–Wallis + Dunn´s post-hoc). Low-fat diet (**LFD**), high-fat diet rich in saturated fatty acids (**HFD-SFA**), high-fat diet rich in olive oil (**HFD-OO**), and high-fat diet rich in olive oil plus DHA and EPA (**HFD-OO-ω3**).

Haemorrhagic transformation was evaluated 48 h after reperfusion in order to determine whether HFDs affected haemorrhagic damage. Haemorrhagic infarction type 1 (HI-1) and 2 (HI-2) or no haemorrhages were found indistinctly in the brain slices from the four experimental groups of mice (Figure 6A,B). None of the animals showed parenchymal haemorrhages type 1 (PH-1) or 2 (PH-2). The analysis of the Haemorrhagic transformation score (HT) revealed that no significant differences between the experimental groups were evident (Figure 6B).

### 3.6. Improvement of Post-Ischemic Neurobehavioural and Physical Outcomes in Mice Fed with Diets Rich in MUFAs from Olive Oil or in DHA/EPA + MUFAs from Olive Oil

To determine the effects of the dietary intervention with HFD-SFA, HFD-OO, or HFD-OO-ω3 diets on brain function and general welfare in mice subjected to focal cerebral ischemia, several functional tests were performed, measuring as grip strength, neurological scale, footprint, and body-weight loss.

#### 3.6.1. Grip Strength Test and Neurological Score

**Figure 7:**
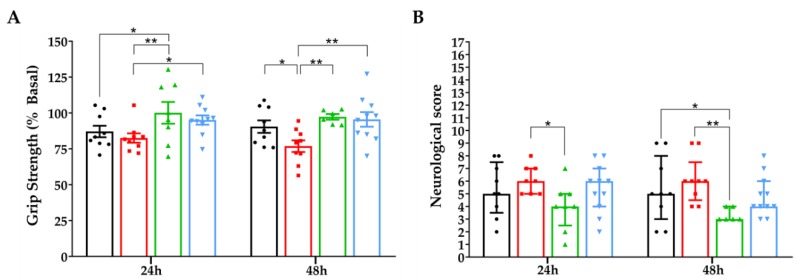
Differences in motor deficits and neurological function in mice fed with HFD-OO or HFD-OO-ω3. Groups: LFD (Black), HFD-SFA (Red), HFD-OO (Green), and HFD-OO-ω3 (Blue). (**A**) HFD-OO and HFD-OO-ω3 diets improved grip strength 24 and 48 h after reperfusion relative to the LFD or HFD-SFA. Mice belonging to HFD-SFA group displayed less grip strength 48 h after the onset of tMCAo than animals on LFD. (**B**) HFD-OO-fed mice had better neurological scores 24 and 48 h after reperfusion compared to HFD-SFA-fed or the HFD-SFA and LFD-fed groups, respectively. The results represent the mean ± SEM in A and the median ± interquartile range in B of 6 to 11 animals: * *p* ≤ 0.05 and ** *p* ≤ 0.01 (ANOVA + Bonferroni post-hoc were performed in (**A**) and Kruskal–Wallis + Dunn´s tests were performed in (**B**)).

Motor and neurological deficits were analysed in animals fed with LFD, HFD-SFA, HFD-OO, or HFD-OO-ω3 diets using a grip strength test and an adapted neurological scale, evaluating the animals before surgery, and 24 h and 48 h after the onset of tMCAo (Figure 7). The functional outcomes indicated better forelimb muscular strength in HFD-OO or HFD-OO-ω3-fed animals compared to HFD-SFA or LFD-fed animals 24 h after tMCAo. In addition, the HFD-SFA-fed animals were the weakest after 48 h relative to the animals fed with HFD-OO, HFD-OO-ω3, or LFD (Figure 7A). Importantly, there were no significant differences in grip strength between each group before the induction of cerebral ischemia (127.5 ± 3.7, 128.5 ± 5.6, 120.4 ± 4.4, and 120.0 ± 3.7 (g) LFD, HFD-SFA, HFD-OO, and HFD-OO-ω3 respectively (mean ± SEM); *n* = 9–12, *p* = 0.36 (ANOVA)). In addition, the neurological scores of HFD-OO-fed mice were better at 24 and 48 h than those fed with HFD-SFA or LFD (Figure 7B). The assessment of neurological function also revealed that the animals fed with HFDs or LFD had similar neurological scores before the induction of tMCAo (0 (0–0.75), 1 (0–1.5), 1 (0–1), and 0.5 (0–1.75 points) LFD, HFD-SFA, HFD-OO, and HFD-OO-ω3 respectively (median and interquartile range); *n* = 9–12, *p* = 0.21 (Kruskal-Wallis)).

#### 3.6.2. Gait Analysis and Weight Loss

**Figure 8:**
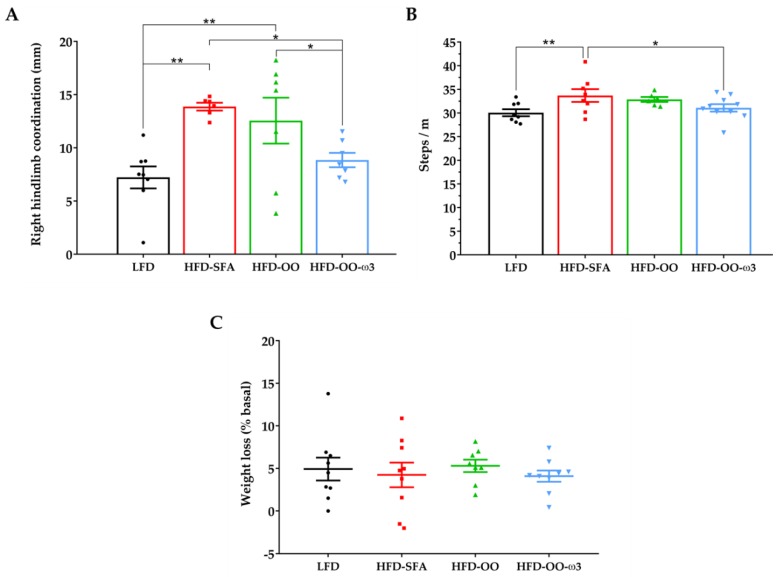
The effect of dietary interventions with a HFD containing a specific fatty acid composition on gait coordination and weight loss. Groups: LFD (Black), HFD-SFA (Red), HFD-OO (Green), and HFD-OO-ω3 (Blue). (**A**) Mice on a HFD-OO-ω3 and LFD diet had better coordination of the right hindlimb 48 h after reperfusion than those on a HFD-SFA diet. (**B**) Mice fed with HFD-OO-ω3 or LFD showed better steps/m values than mice fed a HFD-SFA diet. HFD-OO was not as beneficial as HFD-OO-ω3 48 h after the onset of reperfusion. (**C**) When the weight loss was analysed 48 h after tMCAo, no significant differences were found between the groups. The results represent the mean ± SEM of 6 to 10 mice: * *p* ≤ 0.05 and ** *p* ≤ 0.01 (ANOVA + Bonferroni post-hoc). Low-fat diet (**LFD**), high-fat diet rich in saturated fatty acids (**HFD-SFA**), high-fat diet rich in olive oil (**HFD-OO**), and high-fat diet rich in olive oil plus DHA and EPA (**HFD-OO-ω3**).

The footprint test was performed 48 h after the onset of tMCAo to analyse how the HFD with a specific fatty acid composition affected the gait impairment in mice. Several gait parameters were analysed: stride length, front and base width, overlap, stance, steps/m, and stride coordination. HFD-OO-ω3 and LFD-fed mice had better coordination of the paretic right hindlimb than HFD-SFA-fed mice (Figure 8A). Moreover, mice fed with HFD-OO-ω3 and LFD took fewer steps per meter (steps/m) than animals fed with a HFD-SFA diet (Figure 8B). Interestingly, animals fed with HFD-OO did not show any improvement in any of these parameters compared to the rest of the groups. These results indicate that HFD enriched in DHA/EPA and olive oil, and low-fat diets, can diminish the gait impairment in animals that suffer cerebral ischemia, as opposed to HFD-SFA-fed mice. In addition, we analysed body weight as an indicator of the general welfare of the mice 48 h after tMCAo (Figure 8C). Thus, all animals subjected to focal cerebral ischemia experienced a 5% weight loss, with no significant differences between the groups.

### 3.7. Effect of Custom HFDs on the Antioxidant Enzyme Activities in the Plasma

The activities of antioxidant enzymes catalase (CAT), glutathione reductase (GR), and glutathione peroxidase (GPx) were assessed 48 h after the induction of cerebral ischemia in the plasma from mice fed with LFD, HFD-SFA, HFD-OO, or HFD-OO-ω3. CAT converts the hydrogen peroxide formed in injured tissues to oxygen and water. The activity of this antioxidant enzyme was significantly higher in mice fed HFD-OO (Table 4). GPx reduces lipid peroxides to lipid alcohols and hydrogen peroxide to water, and GR catalyses the reduction of glutathione disulphide (GSSG) to the sulfhydryl form of glutathione (GSH), which plays an important role in oxidative stress and preserves redox homeostasis in cells. We analysed the activity of GR and GPx in the plasma from mice fed with LFD, HFD-SFA, HFD-OO, or HFD-OO-ω3, yet no significant differences between mice were found (Table 4). However, the mice fed with HFD-OO and LFD had a higher GR/GPx ratio than mice fed with HFD-SFA, an indicator of the status of the glutathione redox system (Table 4).

## 4. Discussion

In this study, we demonstrated that dietary fat composition influences the evolution of cerebral injury after tMCAo in mice. The beneficial effects of olive oil and omega-3 PUFA dietary supplementation were evident, both histologically and in neurological behaviour. We showed that a 4-week administration of a high-fat diet enriched in DHA/EPA and olive oil (HFD-OO-ω3) significantly reduces the infarct area and infarct severity in mice subjected to focal cerebral ischemia. Moreover, a HFD-OO-ω3 diet ameliorates the motor deficits and gait disturbance in these animals. The data also show that the administration of a high-fat diet enriched in olive oil (HFD-OO) prior to cerebral ischemia protects against the ensuing neurological and motor deficits in mice. We reported an improvement in the evolution of cerebral damage and in neurocognitive function through the administration of diets enriched in olive oil and omega-3 long-chain fatty acids, reflecting the importance of the type of fat in the diet. These findings confirm the importance of the presence of omega-3 EPA and DHA fatty acids in the diet.

In recent years, the MUFAs and PUFAs present in olive oil and fish oil have been considered to be compounds with neuroprotective effects, particularly against the brain damage associated with stroke. The effectiveness of administering these fatty acids in models of brain ischemia prior to the ischemic insult has been reported in several studies [22,42,43,44]. Specifically, neuroprotective effects have also been seen in animals subjected to cerebral ischemia that were pre-treated with DHA [45,46], EPA [43,47], or fish oil containing both these omega-3 fatty acids [44,48]. In addition, recent studies have shown the beneficial effects of pre-treatment with olive oil, reducing infarct size and improving neurological outcomes in experimental models of brain ischemia [21,22]. In line with these studies, our results show that maintaining a diet enriched in DHA/EPA and olive oil reduced the brain damage and ameliorated the neurological deficit produced by ischemic insult, indicating the importance of dietary fat composition on the damage produced by stroke. We also show for the first time, to the best of our knowledge, that the combination of olive oil and omega-3 fatty acids has a positive effect in a model of experimental stroke.

High-fat diets rich in SFAs are associated with a poor outcome in animal models of cerebral ischemia [7,49,50]. Long-term intake of a HFD before traumatic brain injury altered the effects on cognitive function and the recovery sensorimotor activities, and it was associated with a greater loss of cortical tissue after the insult [11]. Even a short-term HFD without obesity or the induction of a metabolic imbalance through the diet may be detrimental to brain vasculature, producing an exacerbated response to cerebral ischemia, particularly in terms of functional outcomes [14]. Similar effects of HFD-SFA diets were observed under our experimental conditions, with a worse outcome in grip strength and gait after lesion in mice fed with a HFD-SFA than in mice fed a low-fat diet (LFD). However, the motor deficits observed in the HFD-SFA mice were in most cases reversed when SFAs were reduced by partial substitution with PUFAs (DHA/EPA) and MUFAs (from olive oil). In addition, mice fed with HFD enriched in MUFAs from olive oil also produced better neurological scores and performed better in the grip test than mice in the HFD-SFA and LFD groups. Taken together, these results indicate the beneficial effects of the partial substitution of SFAs with DHA/EPA and MUFAs from olive oil in mice subjected to cerebral ischemia.

There are additional consequences linked to HFD consumption, such as the induction of systemic inflammatory responses. High-fat diets are characterized by a high omega-6/omega-3 ratio and it is well-known that a higher intake of omega-6 fatty acids may induce a proinflammatory state [51]. Several studies have demonstrated that stroke patients generally maintain an unhealthy diet, and this is an important factor in the poor outcome of ischemic stroke [6,7,14]. Moreover, a worse outcome in patients following ischemic stroke has been related to neuroinflammation [6,7].

We consider that the composition of dietary fat may influence and modulate the outcome of stroke and the patient’s subsequent evolution by regulating antioxidant and anti-inflammatories activities. Olive oil has antioxidant and neuroprotective properties [52], and recent studies demonstrated the positive properties of DHA treatment after MCAo in rat stroke models, producing a reduction in infarct volume and edema, and an improvement in the neurological deficits due to the activation of NPD1 synthesis [53,54]. Furthermore, DHA reduces apoptosis in the brain by stimulating antiapoptotic processes, dampening responses to ROS, downregulating the expression of proapoptotic proteins, upregulating the expression of antiapoptotic proteins, and preserving mitochondrial function and integrity [55]. Combining multiple agents that target different cellular pathways could maximize the suppression of ischemic signalling, as we have shown in this study, producing additive or synergic effects and improving the outcomes after cerebral ischemia.

A healthy diet is an important part of stroke prevention [56]. International guidelines recommend the reduction of saturated fats, cholesterol intake (such as butter, cream), and high salt diets to prevent vascular risks [1]. Nevertheless, diets enriched in bioactive compounds may also help to reduce brain damage when a stroke occurs. As we have shown here, the consumption of a diet rich in olive oil and omega-3 fatty acids before the ischemic insult occurs may protect the brain and minimize the damage produced. This strategy could be a healthy recommendation especially for patients at high risk of a stroke. Indeed, this type of diet could be a candidate to prove what we proposed previously, a novel alternative approach referred to as advanced neuroprotection strategy [57]. Further research will be required to explore the benefits of this approach.

We should also mention the limitations of this study, such as evaluating the outcome in the acute phase (24–48 h) and only using male mice. Further studies are warranted to explore the underlying mechanisms and to define which type of dietary fat improves short and long-term stroke outcomes in both female and male mouse models, in different strains, or when faced with co-morbid conditions, as well as in aged mice that might better resemble stroke patient profiles.

## 5. Conclusions

This work provides novel evidence of the early impact of a HFD enriched in olive oil and omega-3 fatty acids on the outcomes and neurological function after stroke.

## Figures and Tables

**Table 1:** Macronutrient composition of diets.

Macronutrient	LFD	HFD-SFA	HFD-OO	HFD-OO-ω3
Fat (%)	3	24	24	24
Carbohydrate (%)	77.5	56.5	56.5	56.5
Protein (%)	19.5	19.5	19.5	19.5

Values are expressed as the percentage of energy derived from fat, carbohydrate or protein. Low-fat diet (**LFD**), high-fat diet rich in saturated fatty acids (**HFD-SFA**), high-fat diet rich in olive oil (**HFD-OO**), and high-fat diet rich in olive oil plus DHA and EPA (**HFD-OO-ω3**).

**Table 2:** Fatty acid composition of dietary fats for corresponding high-fat diet (HFD).

	HFD-SFA	HFD-OO	HFD-OO-ω3
Fatty Acid	g/100 g of Fatty Acid		
4:0, butyric	0.83 ± 0.09		
6:0, caproic	0.25 ± 0.01		
8:0, caprylic	0.61 ± 0.04		
10:0, capric	2.47 ± 0.08		
12:0, lauric	3.09 ± 0.24		
14:0, myristic	10.9 ± 0.53		
16:0, palmitic	35.5 ± 0.47	20.4 ± 0.51	20.5 ± 0.37
16:1(ω-7), palmitoleic	3.60 ± 0.18	0.97 ± 0.10	0.82 ± 0.07
18:0, stearic	11.5 ± 0.43	5.70 ± 0.06	4.49 ± 0.21
18:1(ω-9), oleic	25.3 ± 0.41	61.9 ± 0.71	61.5 ± 0.56
18:2(ω-6), linoleic	4.27 ± 0.47	7.97 ± 0.38	8.04 ± 0.31
18:3(ω-3), α-linolenic	0.39 ± 0.03	1.04 ± 0.08	0.94 ± 0.02
20:5(ω-3), eicosapentaenoic			0.92 ± 0.05
22:6(ω-3), docosahexaenoic			0.72 ± 0.06
Others	0.96 ± 0.24	2.05 ± 0.62	2.01 ± 0.51
Total SFAs	63.46 ± 1.07	26.11 ± 0.59***	25.01 ± 0.49***
Total MUFAs	28.93 ± 1.48	62.83 ± 0.78***	62.36 ± 0.59***
Total PUFAs	4.66 ± 0.46	9.01 ± 0.42**	10.62 ± 0.39***

The values are expressed as the mean ± SEM (*n* = 3). An ANOVA followed by a Bonferroni´s post-hoc test was performed: ** *p* ≤ 0.01 and *** *p* ≤ 0.001 versus HFD-SFA. Saturated fatty acids (SFAs), monounsaturated fatty acids (MUFAs), polyunsaturated fatty acids (PUFAs). High-fat diet rich in saturated fatty acids (**HFD-SFA**), high-fat diet rich in olive oil (**HFD-OO**), and high-fat diet rich in olive oil plus DHA and EPA (**HFD-OO-ω3**)**.**

**Table 3:** Individual daily food intake. The results represent the mean ± SEM of 3 to 4 independent animals. No significant differences between the groups were found by one-way analysis of variance (ANOVA).

	Food Intake (g)
**LFD**	4.53 ± 0.20
**HFD-SFA**	5.26 ± 0.25
**HFD-OO**	5.76 ± 0.35
**HFD-OO-ω3**	5.30 ± 0.40

Low-fat diet (**LFD**)**,** high-fat diet rich in saturated fatty acids (**HFD-SFA**)**,** high-fat diet rich in olive oil (**HFD-OO**) and high-fat diet rich in olive oil plus DHA and EPA (**HFD-OO-ω3**).

**Table 4:** Antioxidant enzyme activity in plasma from mice fed with LFD, HFD-SFA, HFD-OO, or HFD-OOω3.

	LFD	HFD-SFA	HFD-OO	HFD-OO-ω3
**CAT** (U/mg protein)	5.53 ± 0.38 ^+^	5.19 ± 0.31 ^+^	8.89 ± 1.92	5.27 ± 0.30 ^+^
**GR** (mU/mg protein)	12.97 ± 1.37	14.02 ± 1.72	16.52 ± 2.61	14.00 ± 1.10
**GPx** (mU/mg protein)	10.29 ± 5.10	33.53 ± 14.62	15.26 ± 5.43	18.86 ± 6.49
**GR/GPx**	1.26 ± 0.29 **	0.42 ± 0.05	1.08 ± 0.23 **	0.77 ± 0.07

CAT, Catalase; GR, glutathione reductase; GPx, glutathione peroxidase. Values are expressed as the mean ± SEM of 3 to 10 animals, subjected to one-way ANOVA followed by Bonferroni´s post-hoc: + *p* ≤ 0.05 versus HFD-OO and ** *p* ≤ 0.01 versus HFD-SFA. Low-fat diet (**LFD**), high-fat diet rich in saturated fatty acids (**HFD-SFA**), high-fat diet rich in olive oil (**HFD-OO**), and high-fat diet rich in olive oil plus DHA and EPA (**HFD-OO-ω3**).
